# Research on Finite Element Model Modification of Carbon Fiber Reinforced Plastic (CFRP) Laminated Structures Based on Correlation Analysis and an Approximate Model

**DOI:** 10.3390/ma12162623

**Published:** 2019-08-17

**Authors:** Yizheng Zhang, Yu’e Yang, Wenhao Du, Qing Han

**Affiliations:** School of Mechanical Engineering, University of Jinan, Jinan 250000, China

**Keywords:** CFRP, Pearson correlation analysis, approximate model, MIGA, finite element model modification

## Abstract

Carbon fiber reinforced plastic (CFRP) laminated structures have been widely used in modern engineering due to their excellent material properties, especially in the aerospace and shipping industries. This requires a high-accuracy finite element model of CFRP laminated structures. However, it is difficult to master the mechanical properties of CFRP structures comprehensively and accurately due to influences from multiple aspects, such as dispersion of material properties, uncertainty of manufacturing technologies, etc. Therefore, a finite element model modification method of CFRP laminated structures based on correlation analysis and an approximate model was proposed. Aiming at minimizing the difference between the analysis model and the measured inherent frequency, the proposed method improves the finite element modeling accuracy of CFRP laminated structures, by iterative optimization based on a global optimization algorithm. In order to solve the problem of high spatial dimension and slow searching in modification of CFRP laminated structure models, the Pearson correlation analysis method was used to screen the material parameters which exert significant impacts on frequency characteristics to reconstruct the searching space. Based on significance parameters, an approximate response model of the CFRP laminated structural system was established. Meanwhile, the modeling accuracy of different orders of response surface models (RSM) and a radial basis function (RBF) neural network model was analyzed, and the best approximate modeling scheme was obtained. The approximate model was updated based on the multi-island genetic algorithm (MIGA) to modify the finite element model of the CFRP laminated structure model. The maximum error and mean error of the updated model are 1.47% and 0.45%. It was proved that the material parameters modified by the proposed method are applicable to simulation analysis of the CFRP laminated structure.

## 1. Introduction

CFRP is a structural composite material with carbon fiber as the reinforcing phase and epoxy resin as the base. Carbon fibers are uniformly distributed in the matrix and bear the most loads, whereas the base bonds the carbon fibers together. In practical engineering structures, CFRP is usually designed as a multi-perspective and multilayer material to make full use of the load bearing of carbon fibers in all directions [1,2,3].

CFRP has obvious directivity because of the influence of material structures. The most common CFRP laminated structure belongs to orthotropic material and its three orthogonal directions have a set of independent material parameters, respectively. The material properties of CFRP are not only related to the composition of fiber and base, but also to the volume content of fibers and interfacial bonding strength. The material parameters may vary with the paving angle and the order of each layer [4,5,6]. Moreover, the performance parameters of CFRP are more dispersed than that of metals, which are related with accuracy of paving angle and interface bonding strength in addition to the multiple components of CFRP [7].

The key of CFRP engineering applications is to find a reasonable CFRP numerical analysis model and grasp the mechanical properties of the CFRP structure comprehensively and accurately. The error between the practical structure and the analysis model cannot be ignored, and this is mainly due to the dispersion of material properties and the uncertainty of the CFRP manufacturing process. Therefore, it is of important engineering significance to study finite element model modification of CFRP laminated structures.

The finite element model modification method can be divided into the matrix-based method and the parameter-based method [8,9,10]. The former directly modifies the mass and stiffness matrixes of the finite element model, without considering the physical significance of parameters in the modified model. This breaks the symmetry and band shape of the original system matrix, and brings great challenges to the subsequent calculations [11].

To prove problems in the matrix-based method, the parameter-based method is proposed for the convenience of engineering applications. The modified results of the parameter-based method have explicit physical significance. The parameter-based method mainly modifies material parameters and geometric parameters, such as the Young modulus, density, sectional area and geometric size of materials [12,13,14]. So far, abundant research on the parameter-based modification method have been proposed in the world, which have achieved outstanding results. For example, Sun et al. [15] tried to decrease error of the finite element model by modifying the equivalent shear modulus of honeycomb sandwich panels. Adel et al. [16] improved the fitting degree between the test results and the simulation analysis results of joint structures by modifying the elasticity modulus of virtual materials of the bolted connecting interface. 

The basic idea of model modification is to minimize the error between the theoretical model and the actual model through optimization [17]. The contributions of parameters to response are generally neglected and a finite element computation is resubmitted after updating all of the involved uncertain parameters at each iteration step in the traditional model modification method. All of these occupy a large amount of computing resources, resulting in low efficiency of the model modification and easy trapping in ill-conditioned problems and non-unique solutions.

To solve the above problems, it is necessary to identify the key parameters which are sensitive to response changes at the early stage of model modification, to effectively reduce the computational cost of high-dimensional models. This is especially necessary for the modification of models involving many uncertain parameters (e.g., CFRP laminated structures). As an effective method to improve model modification efficiency, recognition of key parameters has been widely applied in model modification of various structures. Hernandez-Vazquez et al. [18] screened out the key stiffness parameters for modification of a machine tool model by analyzing the correlations of coupling stiffness of the main components; the coupling stiffness between machine tool and base; and the coupling stiffness of the motor with changes of different orders of frequencies. Huang et al. [19] analyzed the influences of sol-gel delivery pressure, air transportation pressure and replacing speed on degree of haze, glossiness and surface roughness during the coating process of a soda-lime glass surface.

On the other hand, the evocation and calculation of the finite element model during modification is another key factor affecting the modification efficiency of the CFRP laminated structure model. The approximate model method ignores the complexity of the simulation model and establishes an approximate model by fitting input and output data instead of the finite element model. It realizes high-efficiency uncertainty analysis while maintaining a certain accuracy and effectively solves the above problems [20]. Currently, the RSM and RBF neural network models are commonly used approximation models [21,22]. RSM approaches the functional relationship between parameters and response accurately through a simple algebraic expression. With characteristics including a full solid mathematical theoretical basis, strong systematicness and practicability, and a wide application range, RSM has become a powerful tool for the design of complicated engineering systems. Yang et al. [23] revealed the influences of sintering temperature, cold pressure and soaking time on the mechanical properties of a drill by combining RSM and Box–Behnken design. They gained a second-order polynomial equation of mechanical properties. Based on second-order RSM, Boaretti et al. [24] mainly analyzed the influences of technological parameters (such as voltage, distance from probe tip to collector and flow rate), and raw material parameters (degree of sulfonation) on fiber diameter. Based on RSM, Wang et al. [25] discussed the influences of dosage and length of basalt fibers, as well as the asphalt–aggregate ratio on volume and strength of environmentally-friendly basalt fiber-reinforced styrene-butadiene-styrene (SBS)-modified asphalt mixture. 

In theory, high-order RSM also achieves good effects for simulation of complicated problems like nonlinear curved surfaces. However, high-order RSM can significantly increase the calculation workloads with the increase of variables and finally make the calculation costs exceed an affordable level. The RBF neural network model is proposed to offset limitations of RSM and it has the characteristics of quick learning, as well as strong nonlinear approaching ability. Zhao et al. [26] established a functional relationship for influences of volume fraction of nanoparticles in aluminum oxide-water nanofluid, temperature on thermal conductivity, and viscosity based on the RBF neural network. According to prediction results, the prediction errors of RBF in thermal conductivity and viscosity were 0.5177% and 0.5618% respectively, which proved the reliability of predicted results of the RBF neural network. Zhang et al. [27] analyzed the influences of external stress, the plastic zone at crack tip, and crack length on fatigue crack propagation based on the RBF neural network. Djavanroodi et al. [28] studied the neural network model of equal channel angular pressing (ECAP) based on tests and the finite element method, which proved that the feed forward back propagation neural network is applicable to mold design and technology determination for ECAP. Aleksendrić et al. [29] optimized the curing process of thick composite plates based on optimization of an artificial neural network and fuzzy logic controller. Compared with the traditional curing technique, the optimized curing process shortened the curing time by 35% and temperature overshooting was improved by about 10%.

Although model modification technology has been extensively used in various fields, there are few reports on model modification for CFRP laminated structures. In this study, a finite element model modification method of CFRP laminated structures based on correlation analysis and an approximate model was proposed. An orthogonal experimental array of material parameters of CFRP laminated structures was designed, aiming to solve low modification efficiency caused by excessive material parameters and time-consuming finite element iterative computing. Material parameters affecting mostly frequency characteristics were screened by Person correlation analysis. Secondly, a system response approximate model of CFRP laminated structures was constructed based on *c* parameters with significant contributions. Meanwhile, different orders of RSM and RBF neural network modeling accuracies were analyzed in order to obtain the best approximate modeling scheme. Finally, the approximate model was updated by minimizing the error between the analysis model and the test results based on the MIGA. In this way, the finite element model of CFRP laminated structures was modified.

## 2. Theoretical Basis for Model Modification Based on Correlation Analysis and Approximate Model

### 2.1. Model Modification Theory

The basic idea of model modification is similar to the theory of structural optimization. It calculates the dynamic error between the theoretical model and the practical model under the same conditions and then chooses a specific parameter to modify the model to minimize the dynamic error [30]. During modification of CFRP laminated structures mode, it hypothesizes that there are *n* modifying parameters. Then, the overall modifying parameters can be expressed as:

(1) $$X=\left[x_{1},x_{2},x_{3},\ldots ,x_{n}\right].$$

The mass matrix and stiffness matrix of CFRP laminated structures can be expressed as functions of modifying parameters:

(2) $$K=f_{K}\left(X\right),$$

(3) $$M=f_{M}\left(X\right),$$

where *K* is the stiffness matrix, *f_K_* is the mapping function of modification parameters to the stiffness matrix, *M* is the mass matrix, and *f_M_* is the mapping function of modification parameters to the mass matrix.

Obviously, inherent frequency characteristics of a structure are functions of the mass matrix and the stiffness matrix. Therefore, the frequency characteristics of a CFRP laminated structure can be expressed as a function of modifying parameters:

(4) $$f=F\left(K,M\right)=F\left(f_{K}\left(X\right),f_{M}\left(X\right)\right)=F\left(X\right),$$

where *f* is inherent frequency, and *F* is mapping function of modification parameters to inherent frequency.

Finally, the modification of the CFRP laminated structure model can be converted into the following optimization problem. The error between the measured results and the analysis model is minimized by the iterative calculations based on optimization algorithm.

(5) $$\mathrm{minfit}\left(X\right)=\min\overset{n}{\underset{i=1}{\sum }}\left|\frac{f_{i}^{test}\left(X\right)-f_{i}^{FE}\left(X\right)}{f_{i}^{test}\left(X\right)}\right|\;s.t\;VLB\leq X\leq VUB,$$

where *i* is number of modal orders, *n* is total number of modal orders which participate in model modification, *f^test^* and *f^FE^* are test result and finite element calculation results of structural dynamics, and *VLB* and *VUB* distributions represent upper and lower limits of changes of structural design parameters.

### 2.2. Correlation Analysis

Pearson correlation analysis is a statistical approach reflecting the degree of correlation between two data variables. Value of the Pearson correlation coefficient [1, −1] can reflect sensitivity of parameters to response changes. If the Pearson correlation coefficient is 1, the parameters are completely positively correlated with the response changes. If the Pearson correlation coefficient is 0, parameters are independent of the response changes. If the Pearson correlation coefficient is −1, the parameters are completely negatively correlated with the response changes [31]. Degree of correlation between CFRP laminated structural design parameters and inherent frequency is interpreted by the Pearson correlation coefficient. The higher the absolute value of the Pearson correlation coefficient, the stronger the correlation between design variables and inherent frequency.

The Pearson correlation coefficient (*r_k_*) between the response (*y*) and factor *k* is defined as:

(6) $$r_{k}=\frac{\sum_{i=1}^{n}\left(x_{i}-\bar{x}\right)\left(y_{i}-\bar{y}\right)}{\sqrt{\sum_{i=1}^{n}{\left(x_{i}-\bar{x}\right)}^{2}{\left(y_{i}-\bar{y}\right)}^{2}}},$$

where *n* is the number of design points in the test, *x* is the mean of factor *k*, and $\bar{y}$ is the mean of response.

### 2.3. Approximate Model

An approximate model is a method of establishing an empirical model by fitting the relationship between practical design variables and response. In most cases, the fitting relation between design variables and response is unknown. Therefore, it is necessary to select samples within a feasible ranges of factors and seek appropriate factors in selected samples based on test design and numerical analysis. The relationship between these appropriate factors and response should be appropriate.

RSM and RBF neural networks are commonly used as approximation models. In this study, the approximate modeling accuracies of RSM and RBF neural networks for CFRP laminated structures were discussed. The approximate model with the higher accuracy was used to modify the CFRP laminated structure model.

According to the highest order of fitting polynomial, RSM can be divided into first-order, second-order and third-order models. Considering the accuracy and complexity of the model, the second-order RSM is used more frequently. The basic form of second-order RSM is:

(7) $$y=\beta_{0}+\overset{t}{\underset{i=1}{\sum }}\beta_{i}x_{i}+\overset{t}{\underset{i=1}{\sum }}\beta_{ii}x_{i}^{2}+\overset{i\neq j}{\sum }\beta_{ij}x_{i}x_{j},$$

where *y* is the fitting function of response surface, *x_i_* is the design variable *I*, *β_0_* is the constant term of regression model, *β_i_* is the first-order term of the regression model, *β_ii_* is the second-order term of the regression model, and *β_ij_* is the cross term of the regression model.

**Figure 1:**
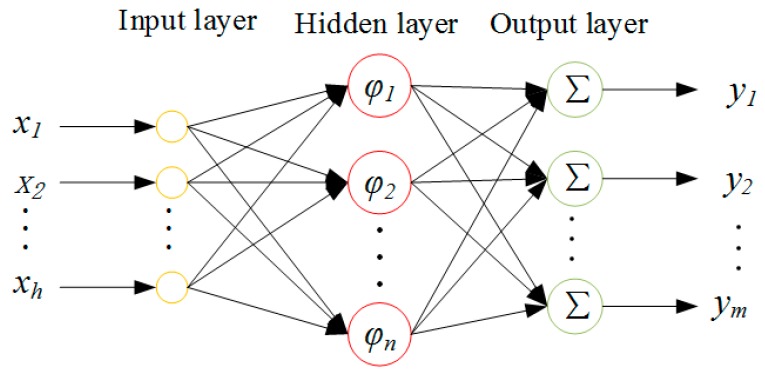
The structure of a radial basis function (RBF) neural network.

An RBF neural network is a kind of feed forward network with single hidden layer. It is composed of an input layer, a hidden layer and an output layer. Topological structure of an RBF neural network with an *h*-*n*-*m* structure is shown in Figure 1. An RBF neural network has a basic characteristic: RBF is used as the excitation function of neurons in the hidden layer, and output layer is the output variable which is gained from linear combination of the hidden layer [32].

Different from RSM, which establishes variables and corresponding relationships, an RBF neural network constructs an approximate model through linear superposition by using the Euclidean distance between sampling data point and measuring point as the variable. The general expression of an RBF neural network is:

(8) $$y=\overset{n}{\underset{i=1}{\sum }}\beta_{i}\phi \left(r_{i}\right),$$

(9) $$r_{i}=\|x-x_{i}\|,$$

where *β_i_* is a weighting factor, *r_i_* reflects the Euclidean distance between the input vector and the design point of test *i*, and *ϕ*(*r*) is the function of Euclidean distance.

### 2.4. Modification Process

Substantially, the modification of the CFRP laminated structure model is an optimization problem. In the present study, an objective function was established based on the inherent frequency error between the analysis model and test results, and the CFRP laminated structure model was modified by the iterative calculation based on the global optimization algorithm. Specific optimization steps are introduced as follows:

1. All 10 material parameters of a CFRP laminated structure are collected through an orthogonal test. The collected data groups are calculated by the finite element method, and the response of the sampling points are obtained. Subsequently, the first material parameters which make the most contributions to changes of inherent frequency are screened by Pearson correlation analysis of all sampling points. These parameters are regarded as model modification parameters.
2. Based on the above six selected material parameters, a new sample space is constructed and the data points needed to construct the approximate model are collected by the optimal Latin square method. Responses of each sampling point are gained through the finite element method. The approximate model is fitted based on the sampling point data and the corresponding responses and model accuracy are tested.
3. The constructed RSM is applied to optimization. The objective function is established between the error between test results and the finite element calculation results of the first four orders of inherent frequency. This objective function is solved by a global searching algorithm (MIGA) and the optimal solution is obtained.
4. The optimized material properties are substituted into the finite element calculation, and the error between the finite element calculation results and the test results of inherent frequency are evaluated after optimization of material parameters. The prediction results of the fifth-order and sixth-order inherent frequencies without modification are analyzed to verify the validity of the model modification.

## 3. Modal Test and Finite Element Analysis

### 3.1. Modal Test

In this section, the modal test of CFRP laminated structures under free boundaries was carried out. The test results were used as the reference values during model modification. In this modal test, CFS-1500 carbon fiber prepregs were processed into a 64-layer CFRP laminated plate in the layering sequence of [45/0/−45/90]_8s_ through hot-pressing technology. The thickness of the 64-layer CFRP laminated plate was 9 mm. Then, the molded 64-layer CFRP laminated plate was cut into 336 mm × 46 mm specimens by a laser engraving machine.

**Figure 2:**
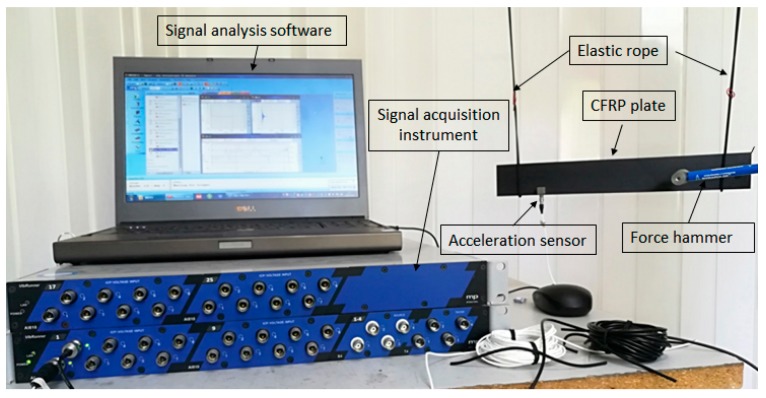
The modal test system.

The single-input single-output (SISO) system was used in the modal test. All specimens were hung by a soft rope to simulate the boundary condition of free vibration. The modal test system was composed of impact equipment, an acceleration sensor, a data acquisition system, and data analysis software (Figure 2). Models of instruments and software used in the modal test system are listed in Table 1.

**Figure 3:**
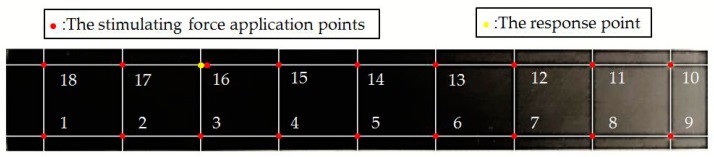
The measuring points arrangement for the carbon fiber reinforced plastic (CFRP) laminated plate.

**Figure 4:**
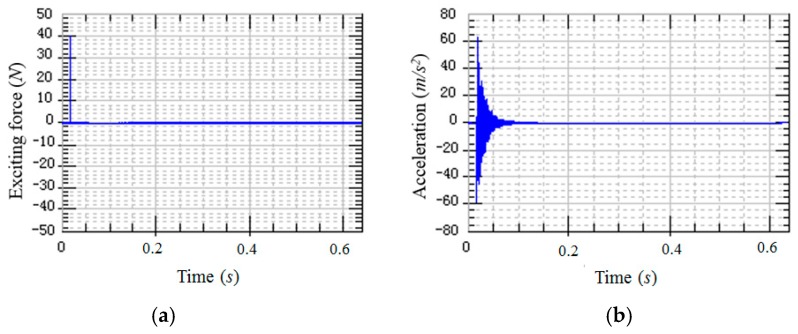
An example of modal test signal. (**a**) Stimulus signal; (**b**) response signal.

The CFRP laminated beam was divided into 18 measuring points (Figure 3). An acceleration sensor was installed as the 16th measuring point to measure vibration response. According to SISO modal test, all 18 measuring points were excited successively by a hammer. Exciting force was measured by the force sensor at the tip of hammer. Response acceleration was measured by an acceleration sensor on the specimen. Typical exciting force signals and response signals are shown in Figure 4. Since the 16th measuring point is occupied by an accelerator sensor, exciting force was applied on the right side of 16th measuring point. Based on linear averaging technique, five effective excitations were applied to every measuring point in order to increase testing accuracy. The average results of frequency domain provide data supports for the follow-up modal analysis.

**Figure 5:**
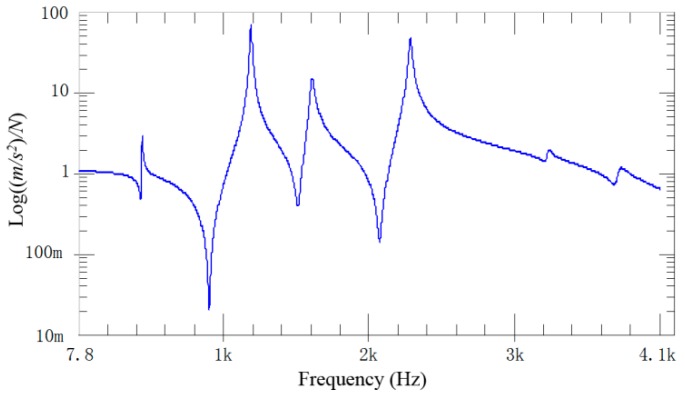
Frequency response function.

Signals were processed and transformed after the time-domain stimulus and response signals were acquired by the signal acquisition system. The frequency response curve reflecting inherent characteristics of the system was calculated by the data analysis software (Figure 5). Each peak of the frequency response curve is corresponding to the inherent frequency point. It can be seen from Figure 5 that a total of six inherent frequencies are identified in the 4 kHz bandwidth.

### 3.2. Finite Element Calculation

**Figure 6:**
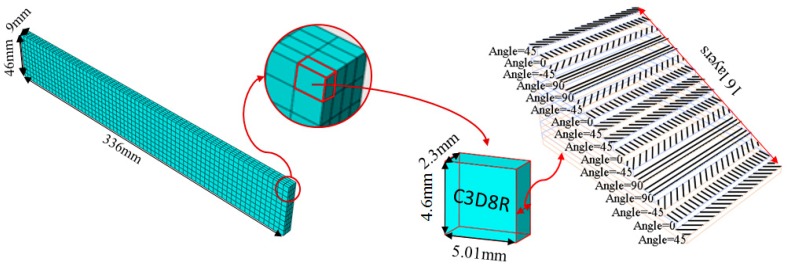
The finite element model of CFRP.

A finite element (FE) model of a CFRP laminated structure was constructed using entity elements with the large universal software ABAQUS2016 to analyze modals. The instruction material parameters of manufacturers are shown in Table 2. The whole CFRP laminated structure was divided into 2680 elements and 3740 nodes through sweep meshing technology and eight-node linear hexahedral C3D8R elements. There were four elements in uniform distribution along the thickness. In each element, 16 layers of fibers were superposed in paving order. Details are shown in Figure 6.

Inherent characteristics of the finite element model were analyzed based on the block Lanczos method under free-free states, which gained the first six orders of bending and torsional modal characteristics (Table 3). As can be seen in Table 3, the six orders include four orders of bending modes and two orders of torsional modes. Although the first order is the first-order bending mode, the bending mode and torsional mode occur alternatively after the second order of mode.

### 3.3. Frequency Contrast Analysis

Low-order inherent frequency has higher test accuracy compared with high-order inherent frequency in modal test results. At present, the finite element model of CFRP laminated structures was modified by the first four orders of inherent frequency values. A quantitative analysis on inherent frequency error (%) between the finite element model and measured results was carried out by calculating the absolute value of the error. The calculation formula of error is:

(10) $$\varepsilon_{i}=\left|\frac{f_{i}^{test}-f_{i}^{FE}}{f_{i}^{test}}\right|\times 100\%,$$

where *f_i_^test^* and *f_i_^FE^* are test results and finite element calculation results of the *i*^th^ order of inherent frequency that represents dynamic characteristics. *ε_i_* is the absolute value of the *i*^th^ order of inherent frequency error (%) between the finite element model and measured results.

Based on analysis of errors in Table 4, the minimum, maximum and mean errors between test results and finite element calculation results are 3.65%, 5.56% and 4.56% which is influenced by dispersion and heterogeneity of CFRP laminated structures. Errors of all orders are higher than the setting value of key structures (<2%–3%) [16]. Therefore, it is necessary to modify the finite element model of CFRP laminated structure to improve the modeling accuracy.

## 4. Model Modification

### 4.1. Pearson Correlation Analysis

Because the CFRP laminated structure involves a large number of material parameters, for optimization they must be determined first, in order to improve model modification efficiency. In this section, the material parameters were selected by an orthogonal test with good space filling performances. Influences of these material parameters on changes of inherent frequency were evaluated by calculating their Pearson correlation coefficients with the inherent response. Material parameters which have great influence on inherent frequency were used as parameters for optimization.

**Figure 7:**
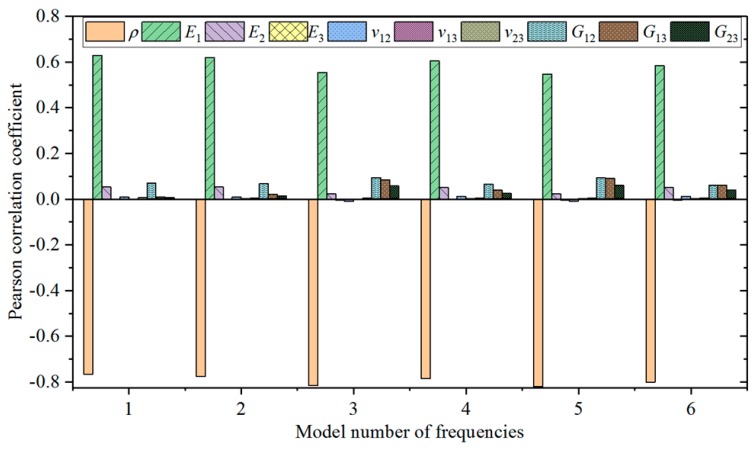
Pearson correlation factors of the CFRP material parameters.

The materials in the CFRP laminated structure are typical anisotropic materials. *E_1_*, *E_2_*, *E_3_*, *G_12_*, *G_13_*, *G_23_*, *u_12_*, *u_13_*, *u_23_* and *ρ* are main material properties. These 10 material parameters were divided into 27 groups of design points through an orthogonal design table L_27_(3^13^) and each parameter was divided into three levels in the designed space. Design points of all material parameters were submitted for finite element calculation, and responses of 27 groups of design points were acquired. Pearson correlation coefficients between material parameters and the first six orders of inherent frequency were calculated according to the Equation (6). The calculated results are shown in Figure 7.

The calculated Pearson correlation coefficients between the first six orders of modal frequency and 10 material parameters of the CFRP laminated structure were analyzed. The results show that density (*ρ*) influences inherent frequency the most, and the Pearson correlation coefficient between density and frequency is negative. Therefore, density shows the most significantly negative correlation with frequency. Elasticity modulus (*E_1_*) along the fiber direction influences frequency the most compared with positively correlated parameters. With comprehensive considerations to influences of parameters on frequency, the first six orders of material parameters with high frequency (*ρ*, *E_1_*, *E_2_*, *G_12_*, *G_13_* and *G_23_*) were selected as parameters for optimization.

**Figure 8:**
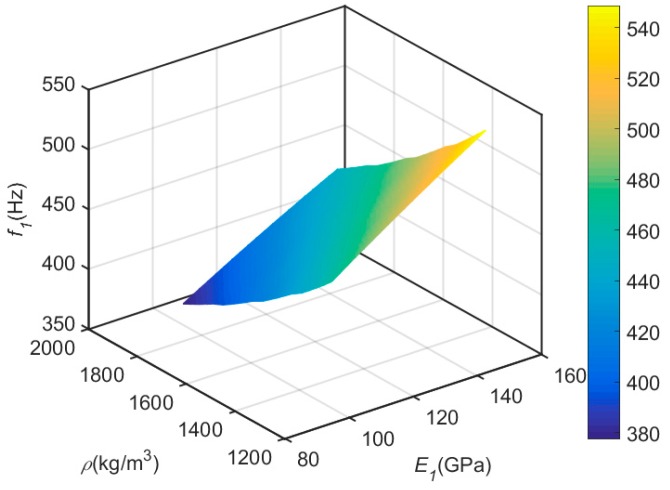
The influencing curved surface of density (*ρ*) and elasticity modulus (*E_1_*) along the fiber direction on first-order inherent frequency (*f_1_*).

Influencing laws of density and elasticity modulus along the fiber direction on inherent frequency were discussed based on the first-order inherent frequency. The influencing curved surface of density (*ρ*) and elasticity modulus (*E_1_*) along the fiber direction on first-order inherent frequency (*f_1_*) was drawn (Figure 8). The first-order inherent frequency has a minimum value at the maximum density and minimum elasticity modulus along the fiber direction. With the decrease of density and increase of elasticity modulus along the fiber direction, value of the first-order inherent frequency increases gradually.

### 4.2. Approximate Model

An approximate model is a method approaching one group of variables and responses by a mathematical model. The approximate models constructed by different approximate modeling technologies have different accuracies. In this section, modeling accuracies of RSM and RBF neural networks in fitting inherent frequency of CFRP laminated structures were discussed. According to the highest number of orders of fitting polynomials, RSM can be further divided into first-order, second-order and third-order RSMs.

Fitting sampling points have to be selected before approximate modeling. In this section, sampling points were selected based on the optimal Latin square method with better homogeneity of sampling factors. Modeling accuracy was assessed by root mean square (RMS) and coefficient of determination (R^2^) of different approximate models. The calculation formulas of RMS and R^2^ are:

(11) $$RMS_{i}=\frac{1}{k{\bar{y}}^{FE}}\sqrt{\overset{k}{\underset{j=1}{\sum }}{\left(y_{ij}^{FE}-y_{ij}^{Ap}\right)}^{2}},$$

(12) $$R_{i}^{2}=1-\frac{\sum_{j=1}^{k}{\left(y_{ij}^{Ap}-{\bar{y}}_{ij}^{FE}\right)}^{2}}{\sum_{j=1}^{k}{\left(y_{ij}^{FE}-{\bar{y}}^{FE}\right)}^{2}},$$

where *k* is total number of optimal Latin square samples, *j* is the total number of sampling points under current calculation, *i* is the number of orders of modals, *y^Ap^* is the output results of the approximate model, *y^FE^* is output of the finite element model, and ${\bar{y}}^{FE}$ is mean output of the finite element model.

In the sample space, *R*^2^ values are between 0 and 1. The fitting function is closer to the finite element calculation results when *R*^2^ is closer to 1, but the fitting functions deviates from the finite element calculation results when *R*^2^ is closer to 0. RMS also values between 0 and 1, but the meaning is opposite to *R*^2^.

**Figure 9:**
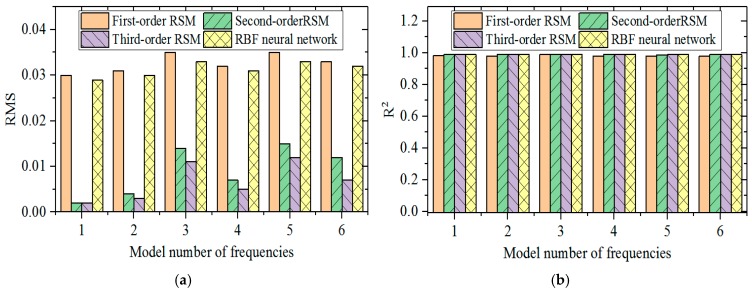
The root mean square (RMS) and coefficient of determination (R^2^) of four approximate models. (**a**) The RMS of four approximate models; (**b**) The R^2^ of four approximate models. Response surface model (RSM).

**Figure 10:**
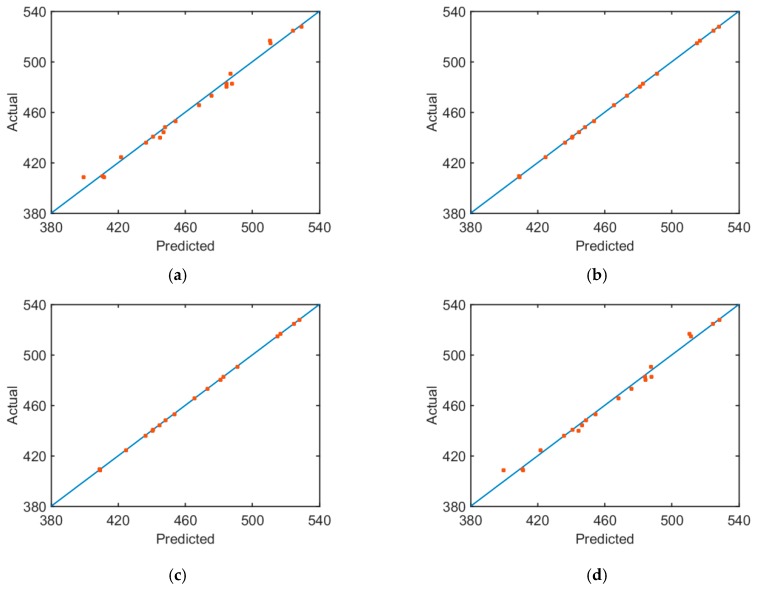
The diagram of the relationship between actual and predicted values of first-order inherent frequencies. (**a**) First-order RMS; (**b**) second-order RMS; (**c**) third-order RMS; (**d**) RBF neural network.

By calculating the RMS and *R*^2^ of different approximate models (Figure 9), it is found that the fitting accuracy of RSM is positively related with the number of orders of the fitting function. Modeling accuracy of the second-order RSM is significantly higher than that of the first-order RSM, but modeling accuracy of the third-order RSM is slightly higher than that of the second-order RSM. The approximate modeling accuracy of the RBF neural network is slightly higher compared with that of the first-order RSM, but it is poorer than modeling accuracies of the second-order and third-order RSM. In order to depict prediction accuracy of approximate models more clearly, the relationship curve between fitting results of four approximate models to the first-order inherent frequency and finite element calculation results is drawn (Figure 10). The degree of deviation of different sampling points from the 45° line can depict approximate modeling accuracy intuitively. If sampling points are concentrated on the 45° line, the approximate model has high modeling accuracy. If sampling points deviate more from the 45° line, the approximate model has lower modeling accuracy.

### 4.3. Updating of Models

In the present study, an objective function was constructed based on the sum of relative errors between test results and approximate model results with respect to the first four orders of inherent frequencies of the CFRP laminated structure. It is expressed as:

(13) $$\mathrm{minfit}\left(X\right)=\min\overset{n}{\underset{i=1}{\sum }}\left|\frac{f_{i}^{test}\left(X\right)-f_{i}^{Ap}\left(X\right)}{f_{i}^{test}\left(X\right)}\right|,$$

where *X* = [*ρ*, *E_1_*, *E_2_*, *G_12_*, *G_13_* and *G_23_*] are modifying parameters. Ranges of modifying parameters fluctuate within 20% based on the original values. *n* = 4 refers to the number of modifying modes. *i* is number of modal orders. *f^test^* and *f^Ap^* are test results and approximate model calculation results of structural dynamics.

**Figure 11:**
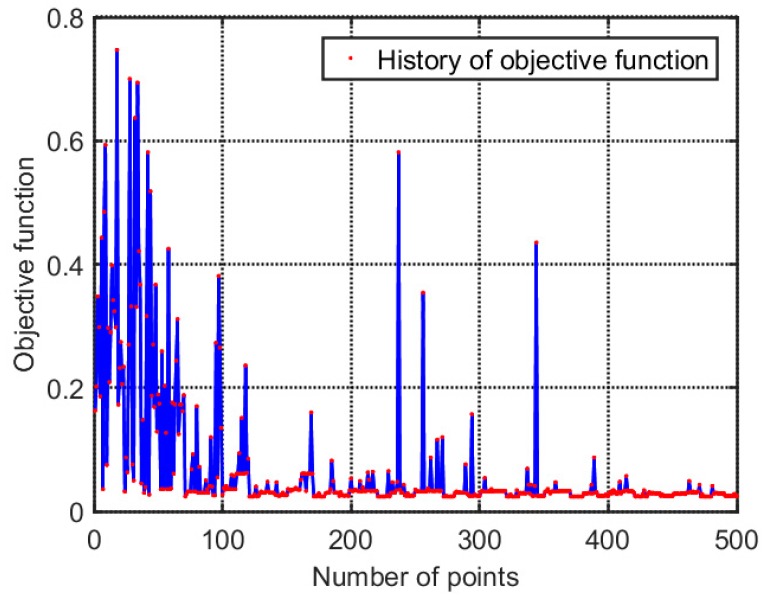
The history of minimizing the objective function.

During model modification, MIGA was used for iterative computing. The parameter settings in MIGA are shown in Table 5. The MIGA stopped at 500 iteration steps. The iteration process is shown in Figure 11. According to the iterative results, the objective function has dispersed results in the first 80 steps and it converges to 0.023 after 80 steps. Although there are oscillations occasionally due to mutation operations of MIGA in the late convergence, the convergence of the optimization results is not affected.

**Figure 12:**
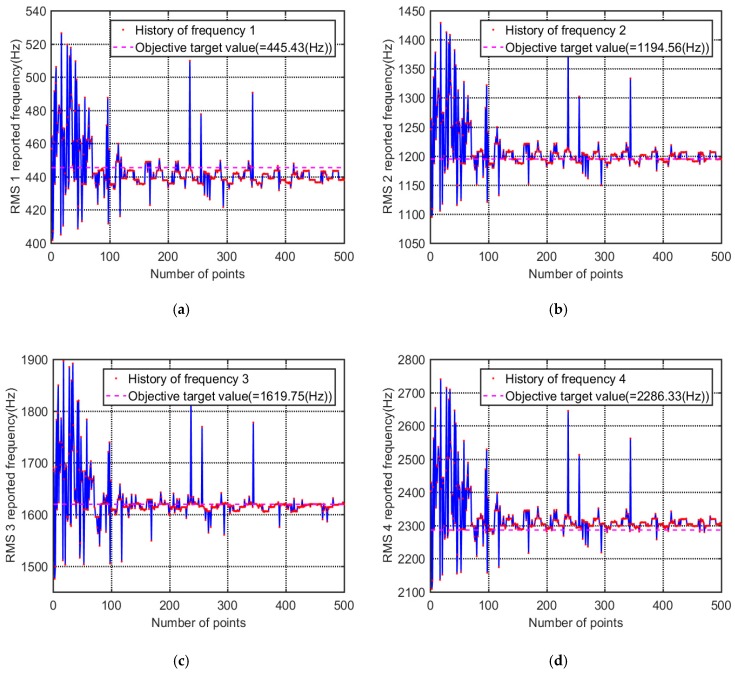
Convergence of inherent frequencies along the updating process. (**a**) First natural frequency; (**b**) second natural frequency; (**c**) third natural frequency; (**d**) forth natural frequency.

Because the modification of the CFRP laminated structure model involves the first four orders of inherent frequency simultaneously, it is a multi-objective optimization problem. In this process, the first four orders of modal frequencies all converge to their respective target values. It can be seen from the iteration curve (Figure 12) that the first four orders of modal frequency show good convergence while protecting the overall objective function.

### 4.4. Verification of Validity

The modified material parameters are shown in Table 6. These modified material parameters were used to replace the initial material parameters of the finite element model and finite element calculation was resubmitted. The first six orders of inherent frequencies after modification are shown in Table 7. According to the analysis of errors in Table 7, the error between the finite element calculation results and test results has been significantly improved after finite element model modification. The maximum and mean absolute values of the error are 1.47% and 0.45%, respectively.

**Figure 13:**
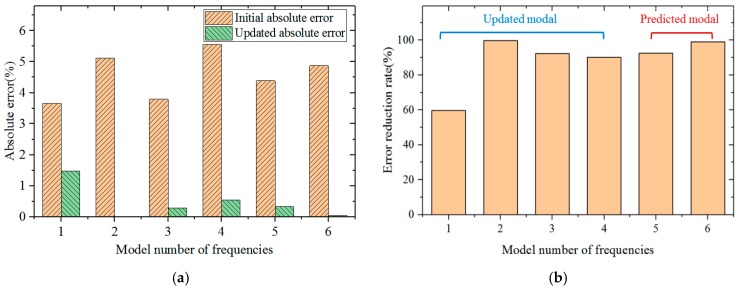
Comparison of error before and after model modification. (**a**) The initial and updated absolute error; (**b**) error reduction rate.

Compared with the initial error, the reduction rates of inherent frequency error of all orders after model modification are shown in Figure 13. The first-order inherent frequency error is decreased by 60% to the minimum extent and the second-order inherent frequency error is decreased by 99% to the maximum extent. The inherent frequency errors of all orders are decreased by more than 90%. For the fifth-order and sixth-order modals which were not modified, absolute values of errors of the prediction modal frequency are only 0.35% and 0.05%, which are 92% and 98% lower than those before modification, respectively.

## 5. Conclusions

The finite element model modification of multilayer CFRP laminated structures based on frequency characteristics was studied using a global optimization algorithm. The error between measured frequency and calculation frequency was shown to be minimized, thus the modification of material parameters in the CFRP laminated structure is realized. To improve modification efficiency, the modifying parameters are screened according to contribution rates to inherent frequency, which further narrows the optimization space and decreases the original 10-dimensional space to a six-dimensional space. As a result, the optimization time is shortened significantly. In addition, the invocation of the finite element model in each iterative optimization step is another important factor that influences modification efficiency. An approximate model is introduced to improve the operation efficiency of the proposed modification method.

Based on calculation of the Pearson coefficient between 10 materials of CFRP laminated structures and inherent frequency, density shows the highest negative correlation with frequency, whereas elasticity modulus along the fiber direction shows the highest positive correlation with frequency. The first six parameters sensitive to changes of inherent frequency are *ρ*, *E_1_*, *E_2_*, *G_12_*, *G_13_* and *G_23_*.

With respect to the approximate modeling problem of inherent frequency characteristics of CFRP laminated structures, although the RBF neural network model is slightly superior to first-order RSM, the calculation accuracy is far lower than that of the high-order RSM model. During comparison of different orders of RSM, the second-order RSM can improve modeling accuracy significantly compared with the first-order RSM, but the third-order RSM actually decreases the modeling accuracy slightly, when compared with the second-order RSM.

The MIGA algorithm shows good convergence characteristics on model modification of CFRP laminated structures. The absolute value of the average error after model modification is only 0.45%, which is far lower than the absolute value of the initial average error (4.56%). Moreover, prediction errors of the fifth and sixth modals, which did not participate in model modification, are only 0.33% and 0.05%, respectively.

## Figures and Tables

**Table 1:** Measurement devices and analysis software employed for modal test.

Name	Type
Signal acquisition instrument	m + p vib runner VR3
Signal analysis software	m + p SO Analyzer
Fore hammer	DYTRAN 5800B4 (sensitivity: 2.25 mv/N
Acceleration sensor	DYTRAN 3097A2 (sensitivity: 98.36 mv/g)

**Table 2:** Mechanical properties of CFRP.

*E_1_*(GPa)	*E_2_* ≈ *E_2_*(GPa)	*G_12_* ≈ *G_13_*(GPa)	*G_23_* (GPa)	*v_12_* ≈ *v_13_*	*v_23_*	*ρ*(kg/m^−3^)
120	10.5	5.25	3.48	0.3	0.45	1520

**Table 3:** The modal shape from experiment and FE.

	Mode Shape from Test	Mode Shape from FE	Description
Mode 1	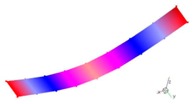	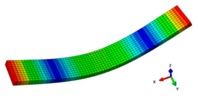	The first bending mode (Bending along y-axis)
Mode 2	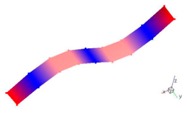	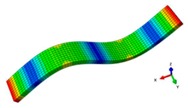	The second bending mode (Bending along y-axis)
Mode 3	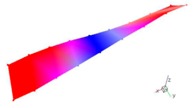	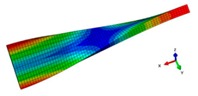	The first torsion mode (Translation along x-axis)
Mode 4	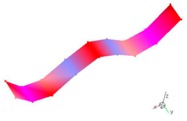	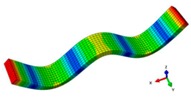	The third bending mode (Bending along y-axis)
Mode 5	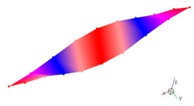	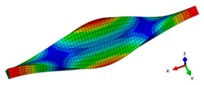	The second torsion mode (Translation along x-axis)
Mode 6	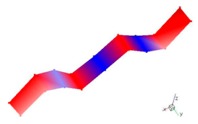	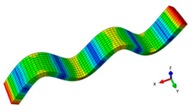	The forth bending mode (Bending along y-axis)

**Table 4:** The inherent frequencies from test and initial FE model.

Mode	Test Frequency (Hz)	Initial FE Model Frequency (Hz)	Absolute Error (%)
1	445.43	461.70	3.65
2	1194.56	1255.7	5.12
3	1619.75	1681.1	3.79
4	2286.33	2413.4	5.56
5	3235.93	3377.9	4.39
6	3707.16	3887.5	4.87
Average value	-	-	4.56

**Table 5:** Parameter settings in the MIGA.

Technical Parameters	Value
Sub-population size	5
Number of islands	5
Number of generations	10
Rate of crossover	1
Rate of mutation	0.01
Rate of migration	0.01
Interval of migration	5

**Table 6:** The initial and final values of material parameters.

Parameter	*ρ* (kg/m^−3^)	*E_1_* (GPa)	*E_2_* (GPa)	*G_12_* (GPa)	*G*_13_ (GPa)	*G*_23_ (GPa)
Initial value	1520	120	10.5	5.25	5.25	3.48
Final value	1684	118.11	10.01	6.21	6.22	3.23

**Table 7:** The inherent frequencies from test and final FE model.

Mode	Test Frequency (Hz)	Updated Frequency (Hz)	Absolute Error (%)
1	445.43	438.87	1.47
2	1194.56	1194.7	0.01
3	1619.75	1615.1	0.29
4	2286.33	2298.9	0.55
5	3235.93	3246.5	0.33
6	3707.16	3709.1	0.05
Average value	-	-	0.45
